# P080 Relapsing giant cell arteritis: atypical relapses, pre-existing osteoporosis and emerging rheumatoid arthritis

**DOI:** 10.1093/rap/rkag113.110

**Published:** 2026-11-03

**Authors:** Amanee Rajapaksha, Jon King, Ognjenka Savanovic-Abel, Thet San, Diana Maria Artene

**Affiliations:** Rheumatology, University Hospitals Plymouth NHS Trust, Plymouth, United Kingdom; Rheumatology, University Hospitals Plymouth NHS Trust, Plymouth, United Kingdom; Rheumatology, University Hospitals Plymouth NHS Trust, Plymouth, United Kingdom; Rheumatology, University Hospitals Plymouth NHS Trust, Plymouth, United Kingdom; Rheumatology, University Hospitals Plymouth NHS Trust, Plymouth, United Kingdom

## Abstract

**Introduction:**

Giant cell arteritis (GCA) is the most common systemic vasculitis in older adults and involves both cranial and large vessels. Relapsing disease is a significant clinical challenge and often necessitates prolonged immunosuppression. Management becomes particularly complex when treatment options are limited due to comorbidities, failure of conventional disease-modifying antirheumatic drugs (DMARDs), or treatment intolerance. We describe a patient with relapsing cranial and large-vessel GCA over five years, complicated by osteoporosis present at diagnosis, failure or intolerance of conventional DMARDs, and subsequent development of inflammatory arthritis requiring repeat biologic therapy.

**Case description:**

A woman in her late 60’s was diagnosed with GCA in 2020 following headache and scalp tenderness without visual involvement. Temporal artery ultrasound demonstrated a halo sign in the temporal and carotid arteries, and temporal artery biopsy confirmed the diagnosis. She responded to prednisolone. Baseline bone densitometry performed shortly after diagnosis confirmed osteoporosis, and she subsequently received riserdronate and zoledronic acid later.

In 2021, while receiving prednisolone 4 mg daily, she relapsed with tinnitus, new onset PMR symptoms, rising C-reactive protein (CRP) and thrombocytosis. Positron emission tomography-computed tomography (PET-CT) demonstrated extensive large-vessel vasculitis involving the thoracic and abdominal aorta, carotid, subclavian, axillary and femoral arteries. Methotrexate was commenced.

Persistent constitutional symptoms and rising inflammatory markers in 2022 prompted escalation of treatment to tocilizumab. Clinical remission was achieved, allowing discontinuation of tocilizumab and glucocorticoids in 2023.

In July 2024, she experienced a further relapse characterised by fatigue, tinnitus, ear and neck pain, weight loss, rising CRP and bilateral axillary artery halo signs on ultrasound. Methotrexate was changed to leflunomide, which was subsequently discontinued because of intolerance.

Several months later, she presented with profound fatigue and scalp tenderness. She attributed the pain to a recent biopsy performed for a suspicious skin lesion; however, tenderness extended beyond the incision, raising concern for recurrent GCA. CRP was mildly elevated at 13 mg/L with persistent thrombocytosis. Temporal artery ultrasound demonstrated new bilateral parietal branch halo signs, confirming recurrent cranial GCA. Prednisolone was recommenced. In the subsequent weeks, she developed symmetrical polyarthritis in hands with multiple tender and swollen joints, and polymyalgic-onset rheumatoid arthritis was considered the most likely diagnosis. Given recurrent GCA, pre-existing osteoporosis, inadequate response or intolerance to conventional DMARDs, and the emergence of inflammatory arthritis, weekly subcutaneous tocilizumab was restarted following local and regional multidisciplinary discussion.

**Discussion:**

This case highlights the challenges associated with persistently relapsing GCA. Over five years, the patient experienced multiple relapses involving both cranial and large-vessel territories despite treatment with glucocorticoids, methotrexate and tocilizumab. Relapse occurred during glucocorticoid tapering, despite conventional DMARDs, and following withdrawal of biologic therapy, pointing out the difficulty of achieving remission in some patients. This reflects the heterogeneity of GCA and raises important questions regarding the optimal duration of targeted therapy in patients at high risk of recurrence.

Recognition of relapse was frequently challenging in this case. Clinical manifestations were often non-specific and varied between episodes. Tinnitus, ear pain, fatigue and PMR symptoms repeatedly accompanied disease recurrence. Furthermore, different vascular territories were involved at each relapse, with inflammation demonstrated in different temporal artery branches, carotid, axillary and large vessels on ultrasound and PET imaging. The most recent relapse was particularly difficult to identify because the patient attributed her scalp tenderness to a recent scalp biopsy. This emphasises the importance of maintaining diagnostic vigilance and integrating clinical assessment with vascular imaging when evaluating suspected relapse.

The emergence of polyarthritis was an additional complexity in this case. Alongside biopsy-proven GCA, she later developed polyarthritis, making polymyalgic-onset rheumatoid arthritis a likely diagnosis. Distinguishing between PMR, peripheral manifestations of GCA and primary inflammatory arthritis can be challenging, particularly when symptoms evolve over several years. The emergence of persistent synovitis broadened the differential diagnosis and influenced therapeutic decision-making.

Management was further complicated by osteoporosis identified at the time of GCA diagnosis. This narrowed the therapeutic margin for repeated glucocorticoid exposure increased the need for effective steroid-sparing treatment. However, methotrexate failed to prevent recurrent disease and leflunomide was not tolerated. Reintroduction of tocilizumab therefore provided a pragmatic strategy to manage both recurrent GCA and inflammatory arthritis while minimising cumulative corticosteroid burden.

**Key learning points:**

• Relapse of GCA could be difficult to recognise and can present with non-specific symptoms rather than classical cranial manifestations. In this patient, tinnitus, ear pain, fatigue and PMR symptoms repeatedly heralded disease recurrence. Clinicians should maintain a high index of suspicion when assessing patients with established GCA, even when typical features such as visual symptoms or jaw claudication are absent.

• Patterns of vascular involvement may evolve over time. This patient developed inflammation affecting temporal, carotid, axillary and large vessels at different stages of her disease course. Vascular imaging played a crucial role in confirming relapse and guiding management, highlighting the importance of ultrasound and PET imaging in patients with suspected recurrent disease.

• Alternative explanations for symptoms should be considered carefully but should not prematurely exclude active vasculitis. During her most recent relapse, the patient attributed scalp tenderness to a recent scalp biopsy. Recognising that symptoms extended beyond the surgical site prompted further investigation and ultimately led to the diagnosis of recurrent cranial GCA.

• The subsequent development of inflammatory polyarthritis highlights the diagnostic overlap between GCA, and polymyalgic-onset rheumatoid arthritis. New musculoskeletal symptoms should be carefully reassessed, as they may represent the emergence of an additional inflammatory condition rather than vasculitis relapse alone.

• Finally, osteoporosis present at diagnosis significantly influenced treatment decisions. In patients with pre-existing osteoporosis, repeated glucocorticoid exposure presents a major management challenge, reinforcing the importance of bone protection measures and effective steroid-sparing strategies. Individualised treatment and multidisciplinary decision-making are essential in complex relapsing disease.

